# Poikilodermatische Mycosis fungoides

**DOI:** 10.1007/s00105-025-05547-4

**Published:** 2025-08-08

**Authors:** Silvia Mihalceanu, Ferdinand Toberer

**Affiliations:** Universitäts-Hautklinik Heidelberg, Ruprecht-Karls Universität Heidelberg, Im Neuenheimer Feld 440, 69120 Heidelberg, Deutschland

**Keywords:** Poikiloderma vasculare atrophicans, Parakeratosis variegata, Parapsoriasis lichenoides, Lichen variegates, Parapsoriasis poikilodermique, Poikiloderma vasculare atrophicans, Parakeratosis variegata, Parapsoriasis lichenoides, Lichen variegates, Parapsoriasis poikilodermique

## Abstract

Die poikilodermatische Mycosis fungoides stellt eine seltene Variante der Mycosis fungoides (MF) dar, die sich durch eine charakteristische Trias aus epidermaler Atrophie, netzförmig konfluierenden, erythematösen Papeln oder Plaques und Teleangiektasien auszeichnet. Histologisch lässt sich ein bandförmiges Infiltrat aus epidermotropen, atypischen Lymphozyten nachweisen. Die diagnostische Abgrenzung gegenüber anderen poikilodermatischen Dermatosen erweist sich als anspruchsvoll, da sowohl klinische als auch histopathologische Überschneidungen bestehen. Wir präsentieren den Fall einer 66-jährigen Patientin mit über 6 Jahre langsam progredienten Hautveränderungen am Rumpf, deren definitive Diagnose erst durch serielle Hautbiopsien und immunhistochemische Analysen gesichert werden konnte. Dieser Fall illustriert die diagnostischen Herausforderungen und hebt die Relevanz einer engmaschigen klinisch-pathologischen Korrelation hervor.

## Abstract (en)

Poikilodermatous mycosis fungoides is a rare variant of mycosis fungoides (MF), characterized by a distinctive triad of epidermal atrophy, reticulated coalescing erythematous papules or plaques, and telangiectasia. Histologically, a band-like infiltrate of epidermotropic atypical lymphocytes can be observed. Diagnostic differentiation from other poikilodermatous dermatoses is challenging due to overlapping clinical and histopathological features. We present the case of a 66-year-old woman with slowly progressive cutaneous lesions on the trunk over a period of 6 years, whose definitive diagnosis was only established through serial skin biopsies and immunohistochemical analyses. This case underscores the diagnostic challenges and highlights the importance of close clinicopathological correlation.

## Anamnese

Eine 66-jährige Patientin stellte sich mit asymptomatischen Hautveränderungen am Rumpf vor. Die Läsionen waren initial auf die linke Brust beschränkt und hatten sich über einen Zeitraum von 6 Jahren langsam auch im Bereich des Abdomens sowie am Rücken ausgebreitet. An Vorerkrankungen hatte die Patientin eine koronare Herzerkrankung bei kardiovaskulären Risikofaktoren (arterielle Hypertonie, Hypercholesterinämie), Asthma und Arthrose, anamnestisch bestand zudem seit ca. 2 Jahren eine Leukopenie. Eine B‑Symptomatik wurde verneint.

## Klinischer Befund

**Abb. 1:**
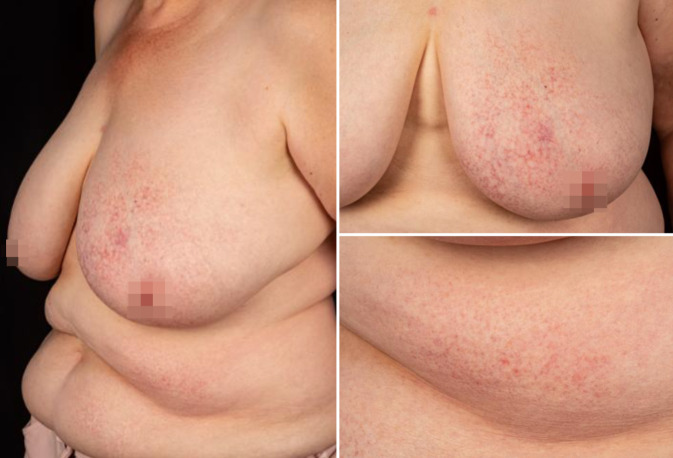
Erythematöse bis violette Makulae und kleine Papeln in einem netzartigen Muster, durchsetzt von Atrophie und auffälligen Teleangiektasien, vorwiegend am Abdomen und der linken Brust

**Abb. 2:**
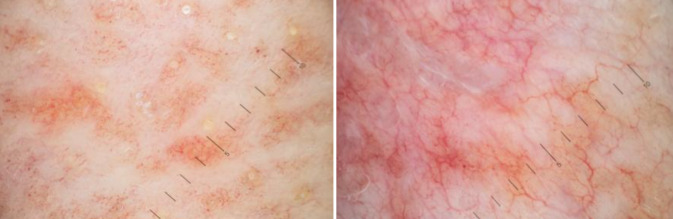
Monomorphes Muster von verzweigten Teleangiektasien auf erythematös-bräunlichem Grund sowie vereinzelt weißliche Schuppung in den auflichtmikroskopischen Aufnahmen

Bei der Inspektion zeigten sich an der linken Brust, am linksseitigen Abdomen sowie weniger ausgeprägt am Rücken und gluteal erythematös bis violette, netzartig konfluierende Papeln, begleitet von Atrophie und auffälligen Teleangiektasien (Abb. 1). Dermatoskopisch zeigten sich ein monomorphes Muster von verzweigten Teleangiektasien auf erythematös-bräunlichem Grund sowie vereinzelt eine weißliche Schuppung (Abb. 2). Die Extremitäten und das Gesicht waren nicht betroffen, ebenso zeigten sich Haare, Nägel und Schleimhäute unauffällig. Die zervikalen, axillären und inguinalen Lymphknoten waren nicht vergrößert palpabel.

## Diagnostik

Eine erste externe histologische Untersuchung hatte differenzialdiagnostisch eine Parapsoriasis en plaque oder ein T‑Zell-Lymphom in Erwägung gezogen. Die in domo initial durchgeführten Hautbiopsien zeigten wiederum eine atrophe lichenoide Dermatitis ohne Epidermotropismus, differenzialdiagnostisch vereinbar mit einer Frühläsion eines Lichen sclerosus et atrophicans oder einem subakut kutanen Lupus erythematodes, und waren aufgrund der Diskrepanz zwischen Klinik und Histologie nicht wegweisend. Erst als sich die Patientin 3 Jahre später wieder vorstellte, konnten wir zusätzliche Hautbiopsien am Rumpf gewinnen, die für die Diagnosesicherung wegweisend waren.

**Abb. 3:**
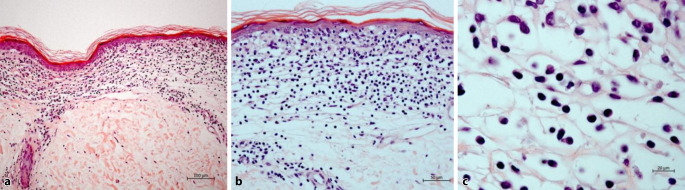
**a** Bandförmiges lymphozytäres Infiltrat mit fokalem „lining up“ von Lymphozyten entlang der Junktionszone unter einer atrophen Epidermis. Weiterhin auffallend dilatierte Kapillaren in einem etwas fibrosierten Bindegewebe (Hämatoxylin-Eosin, Originalvergrößerung 100:1). **b** Vereinzelte Auswanderung der Lymphozyten in das atrophe Epithel (Hämatoxylin-Eosin, Originalvergrößerung 200:1). **c** Bei höherer Vergrößerung imponieren große, atypische Lymphozyten mit entrundeten Kernen (Hämatoxylin-Eosin, Ölimmersion, Originalvergrößerung 630:1)

**Abb. 4:**
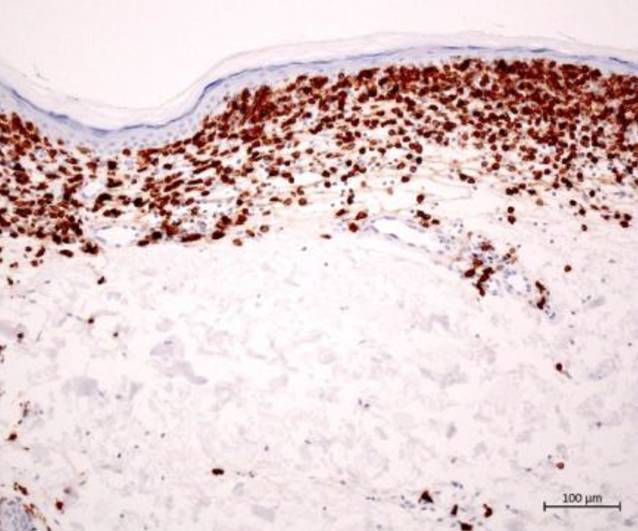
Positive Expression von CD8, Originalvergrößerung 100:1

Histologisch zeigte sich nun eine regelrecht stratifizierte, atrophe Epidermis mit verstrichenen Reteleisten. Darunterliegend zeigten sich ein bandförmiges Infiltrat aus teils atypischen Lymphozyten mit entrundeten Kernen und fokal ein „lining up“ von Lymphozyten entlang der Junktionszone (Abb. 3a, c). Vereinzelt kam auch ein Epidermotropismus zur Darstellung mit Auswandern von Lymphozyten in das atrophe Epithel. Aufliegend imponierte eine kompaktierte Hyperortho- und Parakeratose mit nur wenigen Dyskeratosen, weiterhin auffallend waren dilatierte Kapillaren in einem leicht fibrosierten Bindegewebe (Abb. 3b). Die immunhistochemischen Färbungen ergaben eine Positivität der Lymphozyten für CD3, CD4 und CD8 (Verhältnis CD4:CD8 etwa 2:1) (Abb. 4 und 5), die Färbung mit CD20 zeigte keine nennenswerten eingestreuten B‑Zellen.

**Abb. 5:**
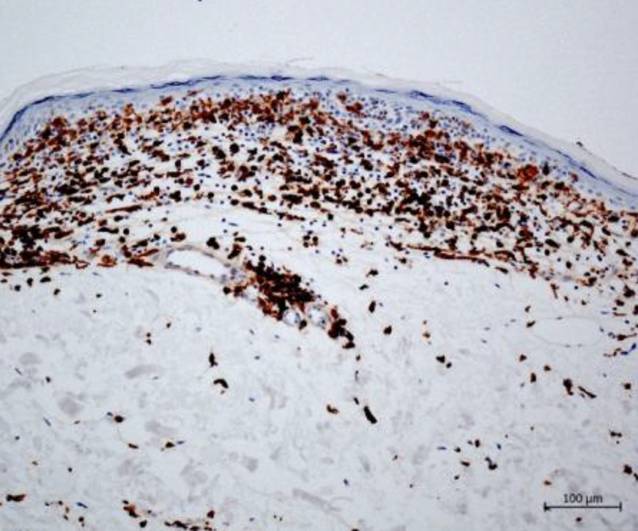
Positive Expression von CD4, Originalvergrößerung 100:1

Bei der molekularen Aufarbeitung des T‑Zell-Rezeptor-gamma-Gens mittels Polymerasekettenreaktion (PCR) zeigte sich eine klonale lymphozytäre Expansion.

Die Korrelation von histomorphologischem, immunhistochemischem und klinischem Bild führte zur Diagnose einer Mycosis fungoides (MF) unter dem klinischen Subtyp der poikilodermatischen Mycosis fungoides. Das Präparat wurde noch in Graz bei Prof. Cerroni zur Mitbeurteilung vorgestellt, hier bestätigte sich die Verdachtsdiagnose. Bei einem Befall von ca. 6 % der Hautoberfläche sowie Abwesenheit von vergrößert palpablen Lymphknoten handelte es sich somit um ein Stadium IA analog der von der ISCL(International Society for Cutaneous Lymphomas)-EORTC(European Organization of Research and Treatment of Cancer)-Klassifikation vorgeschlagenen TNM-Klassifikation der kutanen Lymphome. Sämtliche im Laufe der Diagnostik erfolgten Laboruntersuchungen einschließlich Differenzialblutbild, Serumbiochemie, antinukleärer Antikörper (ANA) und Urinanalyse zeigten sich blande. Die apparative Staging-Diagnostik (Röntgenaufnahme des Thorax, Sonographie des Abdomens und der Lymphknoten) war ebenso unauffällig. Zusätzlich lagen uns extern veranlasste Computertomographieaufnahmen von Brust, Bauch und Beckenorganen vor, die eine paraaortale Lymphadenopathie ohne weitere Auffälligkeiten zeigten.

Die ergänzende FACS(„fluorescence activated cell sorting“)-Analyse zeigte eine deutlich zugunsten der CD4^+^-T-Zellen verschobene CD4:CD8-Ratio von 6,4; 20 % der CD4^+^-T-Zellen wiesen einen CD7-Verlust auf und 13 % einen CD26-Verlust. Unter den T‑Zellen fand sich jedoch keine auffällige Population und insgesamt kein Hinweis auf ein T‑Zell-Lymphom im peripheren Blut.

## Therapie und Verlauf

Bei unserer Patientin erfolgte initial eine topische Therapie mit Mometasonfuroat 1 mg/g Creme 2‑mal täglich für 2 Wochen, dann 1‑mal täglich für 2 Wochen. Hierunter kam es zu einer fast vollständigen Abheilung der Läsionen am Rumpf und einer deutlichen Besserung an der linken Brust. Nach Absetzen zeigte sich wieder ein Rebound der Läsionen, jedoch keine weitere Ausbreitung. Als sich die Patientin erst 3 Jahre später wieder in domo vorstellte, erfolgten aufgrund des benignen Verlaufs und bei fehlenden Hinweisen für eine extrakutane Beteiligung zunächst eine erneute topische Steroidtherapie sowie ein Zyklus (4 Wochen) ambulanter Creme-PUVA(Psoralen mit UV-A)-Therapie. Hierunter zeigte sich bislang ein mäßiges Ansprechen. Da die Hautveränderungen für die Patientin soweit wenig symptomatisch und kaum belastend sind, erfolgte zunächst die bedarfsweise Fortführung der topischen Therapie. Die Patientin wurde zudem dermatoonkologisch an unserem Nationalen Centrum für Tumorerkrankungen angebunden. Geplant ist ein regelmäßiges Follow-up alle 6 Monate für die nächsten 5 Jahre. Eine Therapieeskalation ist bei einer Exazerbation des Befundes vorgesehen.

## Diskussion

Die poikilodermatische Mycosis fungoides ist eine seltene Variante der Mycosis fungoides (MF), gekennzeichnet durch meist generalisiert auftretende, hyperkeratotische schuppige Papeln in netzförmigem oder zebraartigem Muster [1].

In der Literatur werden für dieses Krankheitsbild auch Begriffe wie Poikiloderma vasculare atrophicans (PVA) als Synonyme verwendet. Die Nomenklatur geht auf das Jahr 1890 zurück, als Unna erstmalig lichenoide, retiform angeordnete und konfluierende Papeln begleitet von Epidermisatrophie unter dem Begriff „Parakeratosis variegata“ (PV) beschrieb. Seither wurden ähnliche Hauterkrankungen unter Namen wie Parapsoriasis lichenoides, Lichen variegates, Parapsoriasis poikilodermique, poikilodermale Form der MF oder PVA beschrieben [2].

Die poikilodermatische MF manifestiert sich klinisch in der Regel als kleine rot-braune Papeln oder Plaques mit leichter Schuppung, die zu einem netzartigen Muster konfluieren. Begleitet werden diese oft von Hypo- und Hyperpigmentierungen, Atrophie und Teleangiektasien. Prädilektionsstellen sind die Brust, das Abdomen, die Glutealregion und die Beugen. In der Regel sind Personen mittleren Alters betroffen, wobei Männer etwas überwiegen [3].

Im Gegensatz zur klassischen MF sind poikilodermatische Läsionen im Allgemeinen asymptomatisch oder leicht juckend und in der Regel stabil oder nur langsam größenprogredient [4]. Insbesondere zu Beginn der Erkrankung können die Läsionen dem Lichen ruber planus ähneln.

Differenzialdiagnostisch müssen v. a. Lichen ruber planus, paraneoplastische Poikilodermie, aktinische Poikilodermie, andere Formen der Parapsoriasis sowie bei entsprechender Anamnese eine chronische Graft-versus-Host-Erkrankung abgegrenzt werden. Eine exakte Diagnosestellung erfordert eine Kombination aus klinischer Untersuchung, histopathologischen/immunhistochemischen Kriterien und ggf. molekularbiologischen Analysen [3].

Histologisch zeigt sich ein epidermotrophes Infiltrat atypischer Lymphozyten mit vakuolären Veränderungen in der Basalschicht, meist ohne Bildung von Pautrier-Mikroabszessen in der Epidermis. Charakteristischerweise findet sich auch eine epidermale Atrophie begleitet von Melanininkontinenz und Teleangiektasien. Immunhistochemisch zeigen sich die bandartigen entzündlichen Infiltrate meist positiv für CD8 [5]. Eine Monoklonalität im T‑Zell-Rezeptor-Rearrangement scheint dabei keine Voraussetzung für die Diagnose der poikilodermatischen MF zu sein, zumal ein monoklonales T‑Zell-Infiltrat nicht Lymphom-spezifisch ist. Ebenso schließen das Fehlen von Klonalität oder negative immunhistochemische Ergebnisse die Diagnose MF nicht aus [6, 7]. Die Diagnosestellung kann aufgrund klinischer und histopathologischer Überlappungen mit anderen Erkrankungen herausfordernd sein und bedarf für die definitive Diagnosestellung oft multipler, zeitgleicher Biopsien. Serielle Hautbiopsien sind zudem auch essenziell, um die Entwicklung der lymphozytären Infiltrate über die Zeit verfolgen zu können [4].

Die genaue Ätiologie der poikilodermatischen MF ist nicht abschließend geklärt. Es wird vermutet, dass eine chronische, antigengetriebene T‑Zell-Stimulation eine Rolle spielt. Genetische und immunologische Faktoren sowie Umwelteinflüsse könnten ebenfalls zur Pathogenese beitragen. In einigen Fällen wird eine Assoziation mit chronischen Entzündungen oder viralen Infektionen, insbesondere humanen Retroviren, diskutiert [3].

In der Literatur wird die Zugehörigkeit der poikilodermatischen MF teils noch kontrovers diskutiert. Während einige Arbeiten nahelegen, dass die Erkrankung ein Prämykosid oder eine eigenständige Entität mit distinktem klinischem Verlauf darstellt [3], sind sich die meisten Autoren aufgrund histopathologischer und molekularbiologischer Ähnlichkeiten einig, dass diese klinischen Präsentationen der Mycosis fungoides in einem frühen Stadium zuzuordnen sind.

**Diagnose:** Poikilodermatische Mycosis fungoides

Die Therapie erfolgt in Abhängigkeit vom Erkrankungsstadium. In frühen Stadien orientieren sich die Behandlungsstrategien an der Behandlung der MF im Plaquestadium, wobei topische Kortikosteroide, UV-B- oder PUVA-Therapie sowie topische Retinoide zum Einsatz kommen. Bei progredienten oder therapieresistenten Verläufen können systemische Behandlungen mit Retinoiden (Bexaroten, Acitretin), Interferon‑α oder Methotrexat erforderlich werden. In seltenen Fällen kann eine systemische Chemotherapie notwendig sein [4]. Die Prognose ist insgesamt günstiger als bei der klassischen MF, da die Erkrankung einen langen benignen klinischen Verlauf aufweist und viele Patienten über Jahre stabil bleiben. Dennoch besteht ein geringes Risiko für eine Transformation in eine aggressivere Form des kutanen T‑Zell-Lymphoms, sodass regelmäßige klinische und ggf. histologische Verlaufskontrollen essenziell sind [1].

Zusammenfassend präsentiert die dargestellte Kasuistik eine seltene Form der Mycosis fungoides. Die diagnostische Abklärung basiert entscheidend auf der klinisch-pathologischen Korrelation. Da die histopathologischen Veränderungen insbesondere in frühen Stadien unspezifisch sein können, empfiehlt sich eine längerfristige Beobachtung der Patienten.

## Fazit für die Praxis

- Die poikilodermatische Mycosis fungoides ist eine seltene Variante der Mycosis fungoides und manifestiert sich meist mit asymptomatischen oder leicht juckenden rot-braunen Papeln und Plaques, die zu einem netzartigen Muster konfluieren und von Hypo- und Hyperpigmentierungen, Atrophie und Teleangiektasien begleitet werden. Prädilektionsstellen sind der Rumpf sowie die Gelenkbeugen.
- Histopathologisch zeigt sich ein meist CD8-positives epidermotropes Infiltrat atypischer Lymphozyten mit vakuolären Veränderungen in der Basalschicht, begleitet von epidermaler Atrophie mit Melanininkontinenz und Teleangiektasien.
- Die Zugehörigkeit der poikilodermatischen MF wird in der Literatur zwar noch teils als umstritten beschrieben, jedoch wird diese Entität aktuell als seltene klinische Präsentation der Mycosis fungoides zugeordnet.
- Die Therapie orientiert sich am Stadium der Erkrankung und umfasst in frühen Stadien topische Kortikosteroide, UV-B- oder PUVA(Psoralen mit UV-A)-Therapie sowie topische Retinoide und topisches Chlormetin-Gel. Bei progredienten oder therapieresistenten Verläufen können systemische Behandlungen mit Retinoiden oder Immunsuppressiva erforderlich werden.
- Die Prognose ist insgesamt günstig, dennoch empfiehlt sich eine sorgfältige Nachsorge aufgrund des geringen Risikos einer Progression zu einer aggressiveren Form des kutanen T‑Zell-Lymphoms.
